# The Mechanism for siRNA Transmembrane Assisted by PMAL

**DOI:** 10.3390/molecules23071586

**Published:** 2018-06-29

**Authors:** Yanfei Lu, Jipeng Li, Nan Su, Diannan Lu

**Affiliations:** Department of Chemical Engineering, Tsinghua University, Beijing 100084, China; lu511966207@163.com (Y.L.); lijipeng0927@163.com (J.L.); sunan12345@126.com (N.S.)

**Keywords:** siRNA, PMAL, steered molecular dynamics simulation, PMF, transmembrane delivery

## Abstract

The capacity of silencing genes makes small interfering RNA (siRNA) appealing for curing fatal diseases. However, the naked siRNA is vulnerable to and degraded by endogenous enzymes and is too large and too negatively charged to cross cellular membranes. An effective siRNA carrier, PMAL (poly(maleic anhydride-alt-1-decene) substituted with 3-(dimethylamino) propylamine), has been demonstrated to be able to assist siRNA transmembrane by both experiments and molecular simulation. In the present work, the mechanism of siRNA transmembrane assisted by PMAL was studied using steered molecular dynamics simulations based on the martini coarse-grained model. Here two pulling rates, i.e., 10^−6^ and 10^−5^ nm·ps^−1^, were chosen to imitate the passive and active transport of siRNA, respectively. Potential of mean force (PMF) and interactions among siRNA, PMAL, and lipid bilayer membrane were calculated to describe the energy change during siRNA transmembrane processes at various conditions. It is shown that PMAL-assisted siRNA delivery is in the mode of passive transport. The PMAL can help siRNA insert into lipid bilayer membrane by lowering the energy barrier caused by siRNA and lipid bilayer membrane. PMAL prefers to remain in the lipid bilayer membrane and release siRNA. The above simulations establish a molecular insight of the interaction between siRNA and PMAL and are helpful for the design and applications of new carriers for siRNA delivery.

## 1. Introduction

Small interfering RNA (siRNA) has attracted broad interests in exploring its potential application in gene therapy for fatal diseases, including viral infections and cancers [1,2,3,4]. The naked siRNA, however, is vulnerable to endogenous enzyme and easily degraded. In addition, it is too large (~13 kDa) and too negatively charged to cross cellular membranes. Thus, a safe and effective delivery protocol is pursued for the therapeutic application of siRNA [5,6]. 

Numerous studies have been reported on design of siRNAs and their corresponding carriers, including viral and non-viral siRNA delivery systems [7,8]. Non-viral siRNA delivery carriers—including liposome, lipid-like materials, polymers, and nanoparticles—have been extensively investigated in recent years due to their proven advantages, such as low immune response, safety, and ease of synthesis [9,10]. For example, Grayson et al. have demonstrated the ability of branched polyethylenimide (PEI) of Mw 25000 to deliver siRNA, showing that the delivery efficiency is surprisingly dependent on the biophysical and structural characteristics of PEI [11]. Lee et al. conjugated siRNA to polyethylene glycol (PEG) via disulfide linkage and condensed this conjugates with a cationic fusogenic peptide (KALA) to form nano-sized polyelectrolyte complex micelles, which have an inner core of charge neutralized siRNA/KALA complex surrounded by a PEG corona [12]. In addition, chitosan [13,14], polyarginine [15], curdlan [16], cyclodextrin [17], poly-L-lysine [18], cationic lipids [19], and carbonic nanotube [20] can also be used as carriers for siRNA delivery. 

Among these potential siRNA delivery carriers, amphiphilic polymer materials have arousing concerns due to higher delivery efficiency and safety [21,22,23]. More recently, with an appreciation of the potential of maleic anhydride based copolymers, which is approved by the US FDA, we demonstrated the potential capacity of poly(maleic anhydride-alt-1-decene) substituted with 3-(dimethylamino) propylamine (PMAL) as the siRNA carrier by using steered molecular dynamics simulations and laser scanning confocal microscopy [24]. After that, PMAL is reported to have low protein interaction and high biocompatibility that may be used as a carrier to deliver not only siRNA but also proteins [25].

Molecular simulations have been applied to probe the interactions among polyelectrolytes [26,27,28], including siRNA and synthetic polymers [29]. Ouyang et al. adopted an atomic molecular dynamics simulation to study the complexation of short strand duplex RNA with four cationic carrier systems of varying charge and surface topology at different ratios. It is shown that a dendrimer of higher molecular weight favors a more stable complex with siRNA in low pH [30]. Sun et al. elucidated the role lipid substitution on complexation of PEI and siRNA via all-atom molecular dynamics simulations. They pointed out that the association of lipid side-groups results in more stable and compact PEI/siRNA complex [31]. Karatasos et al. also demonstrated the effect of pH and the size of polyamidoamine dendrimer on its complexation with siRNA [32]. 

In our previous work, we conducted a coarse-grained PMAL model derived from an all-atomic model for the first time and explored how PMAL facilitated the delivery of siRNA [24]. It is shown that the use of PMAL can reduce the energy barrier for siRNA to penetrate lipid bilayer membranes. More importantly, PMAL can punch a hole in the lipid bilayer and form a channel for siRNA delivery. PMAL prefers to remain in lipid bilayers due to hydrophobic interaction between PMAL and lipid molecules and exhibits extended linear structure while siRNA is delivered into the cell [24]. In addition, complexation and dissociation of siRNA and PMAL with different molecular weight has been discussed, which is of fundamental importance for the design of suitable PMAL for siRNA delivery [33]. 

Based on our previous work, further study of the siRNA delivery with PMAL is discussed in this paper. Here we try to elucidate the mechanism of PMAL-assisted siRNA transmembrane via a steered molecular dynamics simulation. First, two possible transmembrane processes, i.e., passive transport and active transport, were modelled by carefully tuning the rate of siRNA or siRNA-PMAL complex through the membrane. Then we studied how the transport behavior affected the interaction between siRNA-PMAL complex and its implication on the structure of complex and lipid bilayer membrane. Finally, we proposed the mechanism of PMAL-assisted siRNA transmembrane in most possible transport modes. 

## 2. Results

### 2.1. Transmembrane Process for the Naked siRNA

**Figure 1:**
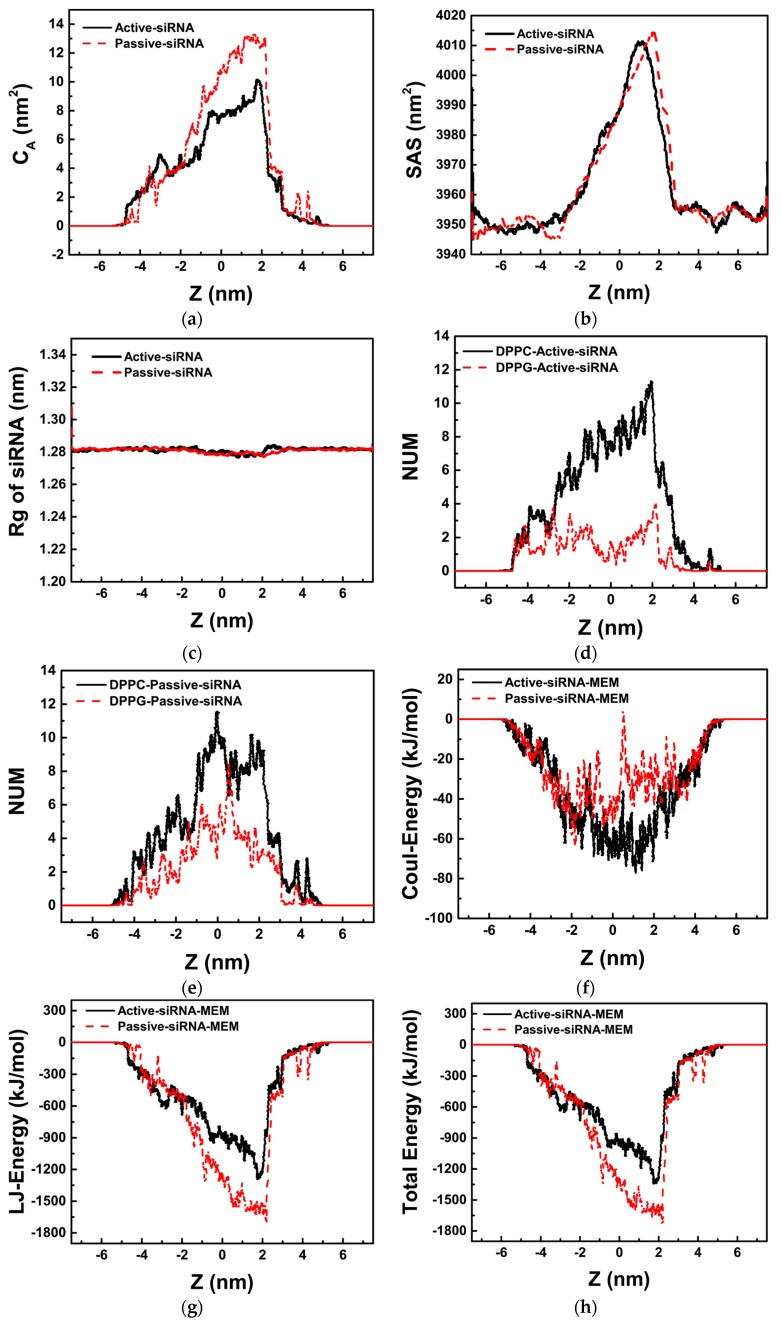

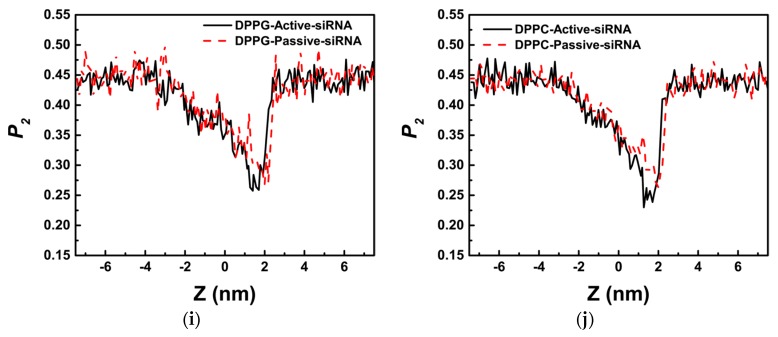
The transmembrane process of the naked siRNA where Z represents the distance between the mass center of siRNA and the center of lipid bilayer membrane along the Z direction as shown in Figure 9d. (**a**) Changes of contact area between siRNA and membrane; (**b**) solvent accessible surface area of lipid bilayer membrane; (**c**) the radius of gyration of the naked siRNA; (**d**) lipid number around the naked siRNA in the case of active transport; (**e**) lipid number around the naked siRNA in the case of passive transport; (**f**) Coulomb interaction energy between the naked siRNA and lipid bilayer membrane; (**g**) L–J interaction energy between the naked siRNA and lipid bilayer membrane; (**h**) total interaction energy between the naked siRNA and lipid bilayer membrane; (**i**) order parameter (*P*_2_) of DPPG molecules in lipid bilayer membrane; (**j**) order parameter (*P*_2_) of DPPC molecules in lipid bilayer membrane.

We firstly studied the naked siRNA at different pulling rates, which are shown in Figure 1. As mentioned above, two pulling rates, 10^−6^ nm·ps^−1^ and 10^−5^ nm·ps^−1^, were chosen to imitate the passive and active transmembrane of the naked siRNA, respectively.

First, we give the change of contact area (*C*_A_) of the naked siRNA and lipid bilayer membrane during transmembrane process, as shown in Figure 1a. It is shown that the contact area increases when the naked siRNA enters the membrane, and reaches highest value when siRNA totally is imbedded into the membrane. After that, the contact area decrease and reaches to zero when siRNA totally departures from the membrane. It is also shown in Figure 1a that the contact area increases to maximum of 12.6 nm^2^ for passive transport process, which is larger than that for active transport process (10 nm^2^), indicating more interaction between siRNA and lipid membrane when the passive transport occurs. 

During transmembrane process, the deformation of lipid bilayer membrane occurs synchronously as shown in Figure 1b. For both the active and passive transport, the surface accessible area of lipid bilayer membrane increases from 3950 nm^2^, which is the value at equilibrium state of free lipid bilayer membrane, to about 4010 nm^2^ when the naked siRNA is totally embedded into membrane. Once the naked siRNA departures totally from membrane, the surface accessible area of lipid bilayer membrane returns to its original equilibrium state. 

It is shown in Figure 1c that the radius of gyration (*R*g) of the naked siRNA maintains 1.28 nm for both the active and passive transport. This is because of rigidness of short siRNA, which is consistent with our previous work [24,33]. Combined with Figure 1b, it is further demonstrated that the changes in solvent accessible surface area is caused by the deformation of lipid bilayer membrane. 

Figure 1d,e give numbers of lipid molecules around siRNA for the active and passive transport of the naked siRNA, respectively. As mentioned, there is three-fold difference in the number of DPPC used to create the lipid bilayer membrane, thus higher contact with DPPC is expected. However, in the active transmembrane of siRNA as shown in Figure 1d, about five-fold difference is obtained, indicating electrostatic interaction between positively charged head group of DPPC and negatively charged siRNA makes more DPPC molecules accumulate around siRNA. Compared with active transport, the number of DPPG molecules around siRNA for the passive transport increases, indicating that DPPG molecules prefer to accumulate in the curvature of the lipid bilayer membrane, which is caused by the naked siRNA. 

We further analyze the interaction energy between the naked siRNA and lipid bilayer membrane as shown in Figure 1f–h. It is shown that the electrostatic interaction energy between siRNA and membrane is significantly lower than the L–J interaction energy. For the passive transport, lipid molecules have enough time to rearrange around siRNA, resulting in more interaction between lipid and siRNA than those for the active transport. This is consistent with result shown in Figure 1a. 

Figure 1i,j give changes of the order parameter during the passive and active transport. It is shown that the values of P_2_ decrease from 0.45 to 0.25 when the naked siRNA is totally embedded into lipid bilayer membrane, indicating the disorder of lipid membrane caused by siRNA. When siRNA departs from membrane, it can return to its intact structure. For both the passive and active transport, DPPG and DPPC molecules have similar behavior. 

**Figure 2:**
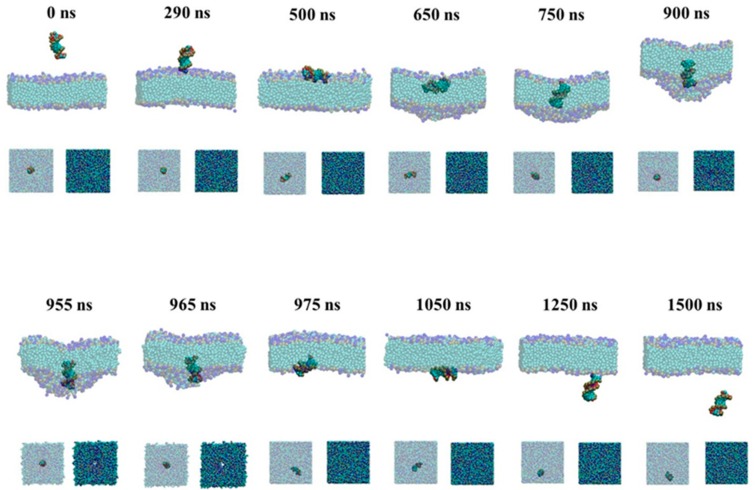
Snapshots of active transmembrane process of the naked siRNA. The major graphs are front view of process and the small graphs are upper view.

**Figure 3:**
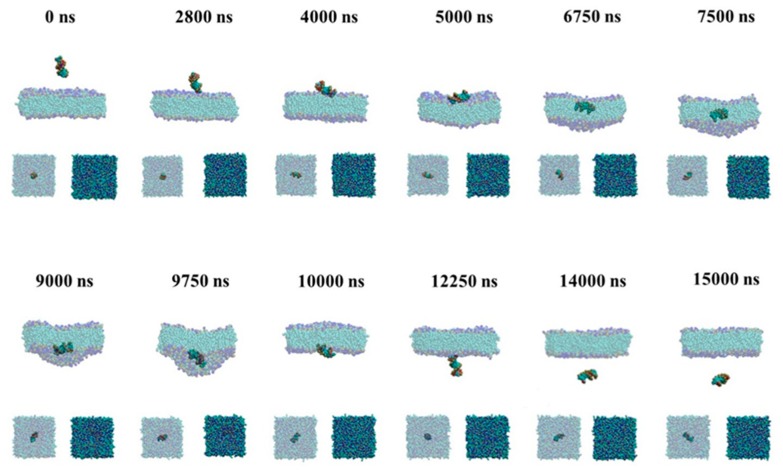
Snapshots of passive transmembrane process of the naked siRNA. The major graphs are front view of process and the small graphs are upper view.

To give the details of the passive and active transport of the naked siRNA through the membrane, we give the snapshots during transmembrane process in Figure 2 and Figure 3.

#### 2.1.1. Active Transport Process for the Naked siRNA

As shown in Figure 2, the naked siRNA moves to the lipid bilayer membrane and touches the membrane surface perpendicularly at 290 ns. Then the naked siRNA lies down and is parallel to the membrane caused by the membrane resistance at 500 ns. When the naked siRNA enters the lipid bilayer membrane, the membrane is caved at 650 ns. Finally, the naked siRNA breaks the upper leaflet of membrane at 750 ns, resulting in totally insertion of siRNA. It should be emphasized here that the naked siRNA prefers inside membrane perpendicularly caused by the rigidity of the naked siRNA and the quick pulling rate. When the naked siRNA further transports through the membrane, the deformation of lipids bilayer is exacerbated at 900 ns. At 955 ns, the naked RNA molecule penetrates the lower leaflet of membrane layer, resulting in the formation of pore at 965 ns. Due to the hydrophilicity of siRNA, water molecules can also penetrate this pore during siRNA delivery. The hydrophilic head groups of those lipid that participate the formation of pore are located at the inner surface of the pore. It indicates the pore is hydrophilic. Then, the naked siRNA can transport through the membrane at 975 ns. At this time, siRNA rotates and lies down again at the surface of lipid bilayer membrane. When the naked siRNA totally departs from the surface of membrane at 1250 ns, the naked siRNA is perpendicular to the membrane again.

#### 2.1.2. Passive Transport Process for Single siRNA

As shown in Figure 3, the passive transmembrane process of the naked siRNA is similar to the active transmembrane process shown in Figure 2. The naked siRNA lies down on the membrane surface and results in the deformation of membrane at 5000 ns. The difference is that the naked siRNA is vertical and inserted in the bilayer in the active transport, while in the passive process the naked siRNA can transport parallelly through the membrane. In the case of passive transport process for single siRNA, the pulling rate applied in the center of siRNA is one-order less than that in the case of active transmembrane. The siRNA embedded into the lipid membrane have enough time to make lipid molecules rearrange around siRNA. This leads to the reduce the force imbalance and make the naked siRNA keep parallel orientation inside the membrane. This results in the naked siRNA exposing to more lipids. Compared with active transport, in which the naked siRNA is vertical to the surface of membrane when it enters, siRNA lies on the surface of membrane and is slowly immersed into membrane in the passive transport. During our simulation, the difference of bending of membrane is not significant. The flatness of lipid membrane shows similar behavior for active and passive process (Figure S6).

### 2.2. Transmembrane Process of siRNA Assisted by PMAL

**Figure 4:**
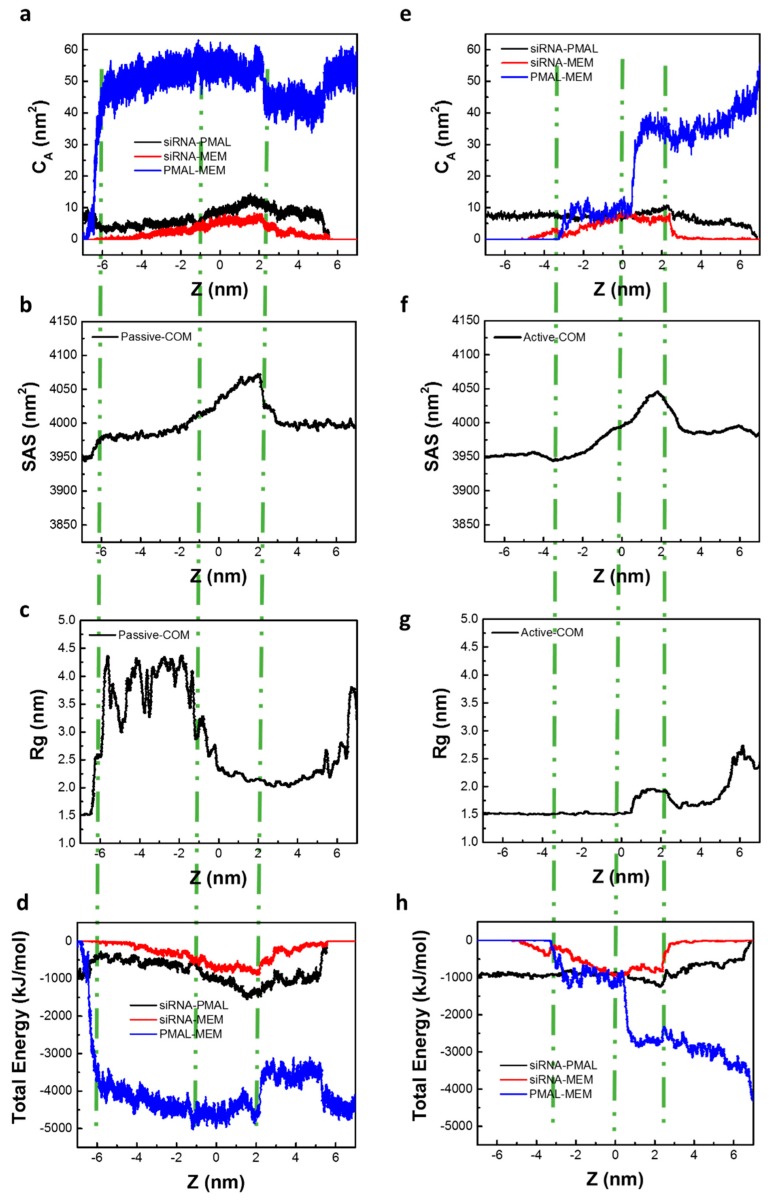
The active and passive transmembrane of siRNA assisted by PMAL where Z represents the distance between the mass center of siRNA and the center of lipid bilayer membrane along the Z direction as shown in Figure 9e. Three green lines from left to right in each figure give the position of siRNA entering, in the center, and leaving the lipid bilayer membrane. (**a**,**e**) The changes of contact area between siRNA and PMAL, siRNA and membrane, as well as PMAL and membrane in the passive and active transport, respectively; (**b**,**f**) The change of solvent accessible surface area of lipid bilayer membrane in the passive and active transport, respectively; (**c**,**g**) the changes of radius of gyrate of PMAL in the passive and active transport, respectively; (**d**,**h**) the changes of total interaction energies among siRNA, PMAL, and membrane in the passive and active transport, respectively.

We also studied the passive and active transport of siRNA assisted by PMAL through the lipid bilayer membrane, which is shown in Figure 4a–h, respectively.

Figure 4a give the changes of contact area in passive transport. It is shown that the contact area between siRNA and PMAL slight decreases when siRNA-PMAL complex enters the lipid bilayer membrane, accompanying a sharp increase of contact area between PMAL and membrane, while the contact area between siRNA and membrane is nearly zero. This indicates that PMAL prefers to interact with membrane, which is consistent with our previous work [24,33]. The interaction between PMAL and membrane results in the deformation of the lipid bilayer membrane, resulting in the increase of surface accessible contact area of membrane as shown in Figure 4b. At the meantime, the interaction between PMAL and membrane results in the increase of radius of gyration of PMAL as shown in Figure 4c, indicating the swelling of PMAL inside lipid bilayer membrane. This is caused by the hydrophobic interaction between lipid tails inside membrane and hydrophobic core of PMAL. The strong interaction between PMAL and lipid bilayer membrane can also be demonstrated by the increase of energy between PMAL and membrane as shown in Figure 4d.

Once the mass center of siRNA is in the middle of lipid bilayer, which is located at Z = −1 nm in the passive transport, the contact area between PMAL and lipid bilayer membrane reaches near 60 nm^2^, indicating the strong interaction between membrane and PMAL. In the meantime, the contact area between siRNA and PMAL and that between siRNA and the membrane only slightly increases (Figure 4a). However, the solvent accessible surface area of membrane still significantly increases as shown in Figure 4b, indicating the destruction of lipid bilayer membrane once the siRNA-PMAL complex is embedded into the membrane. The radius of gyration of PMAL exhibits extended conformation inside membrane as shown in Figure 4c.

When the mass center of siRNA leaves the center of lipid bilayer membrane—i.e., Z ranges from −1.0 nm to 2.0 nm—the contact area between PMAL and lipid bilayer membrane maintains 60 nm^2^ and sharply decrease to 40 nm^2^ when siRNA begins leaving membrane as show in Figure 4a. In the meantime, the solvent accessible surface area of membrane still increases until siRNA begins leaving membrane at Z = 2 nm (Figure 4b). The radius of gyration of PMAL, however, decreases to 2.0 nm (Figure 4c), indicating the dissociation of siRNA-PMAL complex results in the collapse of PMAL inside membrane. The interaction between PMAL and membrane also decreases as shown in Figure 4d.

When siRNA departures from lipid bilayer surface, the contact area between siRNA and membrane and those between siRNA-PMAL decrease to zero, indicating the complete dissociation of siRNA-PMAL complex and siRNA transports through membrane successfully as shown in Figure 4a. While the contact area between PMAL and membrane still maintains from 40 to 60 nm^2^, indicating PMAL remains in lipid bilayer membrane after siRNA delivery. The solvent accessible surface area of membrane recovers to 4000 nm^2^, slightly higher than those of intact lipid bilayer membrane. This indicates the destruction of membrane caused by insert of PMAL. 

In summary, PMAL can interact with siRNA and help it across the membrane and finally release it, but PMAL itself leaves in the membrane in the passive transport of siRNA delivery. 

In the active transport of siRNA assisted by PMAL, however, the interaction between PMAL and lipid bilayer membrane is relatively weaker than that in the passive transport, especially when the siRNA-PMAL complex touches the membrane (Z = −3.4 nm) and moves into the center of membrane (Z = 0 nm) as shown in Figure 4e,g. This indicates that siRNA-PMAL complex does not dissociate because the complex quickly penetrates into the membrane. In the meantime, the lipid bilayer membrane is only slightly deformed, which is demonstrated by the slight increase of solvent accessible surface area shown in Figure 4f. The PMAL maintains intact state with no changes in radius of gyration as shown in Figure 4g.

Once siRNA is delivered from the lipid bilayer membrane (Z = 0→2.2 nm), however, the interaction between PMAL and membrane sharply increases, showing the significant increase of contact area and the decrease of total interaction energy between PMAL and membrane, as shown in Figure 4e,g. In the meantime, the solvent accessible surface area of membrane and radius of gyrate of PMAL both increase, indicating the deformation of lipid bilayer membrane and swelling of PMAL in membrane, respectively.

When siRNA departs from the lipid bilayer surface, the contact area between siRNA and membrane and those between siRNA-PMAL both decrease to zero, indicating the complete dissociation of siRNA-PMAL complex and siRNA transports membrane successfully as shown in Figure 4e. While the contact area between PMAL and membrane continuously increases to 50 nm^2^, indicating PMAL remains in lipid bilayer membrane after siRNA delivery.

Compared to those in the passive transport of siRNA assisted by PMAL, the major difference in active transport is the interaction between PMAL and membrane is weakened, especially when siRNA-PMAL complex enters into the lipid bilayer membrane. This, we believe, is not favorable for the PMAL-assisted siRNA delivery. We will further demonstrate it later by using PMF analysis.

Moreover, the partially disruption of lipid membrane is not avoided during siRNA delivery. The effect of PMAL and siRNA on the membrane is given in Figure S3. As we can see, alignment with the bilayer was disturbed more seriously with PMAL than the naked siRNA, particularly in the passive mode shown in Figure S3a,b. The lipid bilayer membrane is composed of DPPC and DPPG molecules. Similar to the transport of the naked siRNA as shown in Figure 1i,j, the number of DPPC molecules around siRNA is significantly larger than that of DPPG molecules in the active transport (Figure S3c), while the number of DPPG molecules around siRNA increases in the passive transport (Figure S3d). Moreover, the ratio of DPPG and DPPC around PMAL is significantly higher than that in free lipid bilayer membrane (Figure S3e,f). This indicates that PMAL has a greater attraction to DPPG than DPPC due to the electrostatic interaction. Due to the strong interaction between PMAL and DPPG, more DPPG molecular move from upper leaflet to lower leaflet when PMAL moves from the upper leaflet to the lower leaflet during the passive transport of siRNA assisted by PMAL.

**Figure 5:**
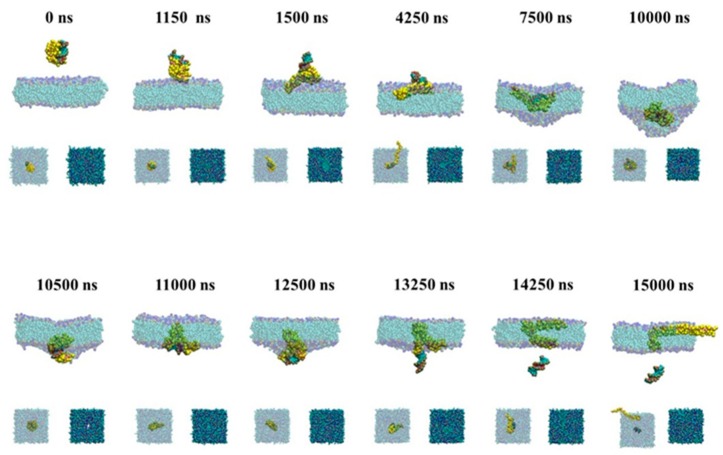
Snapshots of the passive transport of siRNA assisted by PMAL. The major graphs are front view of process and the small graphs are upper view.

**Figure 6:**
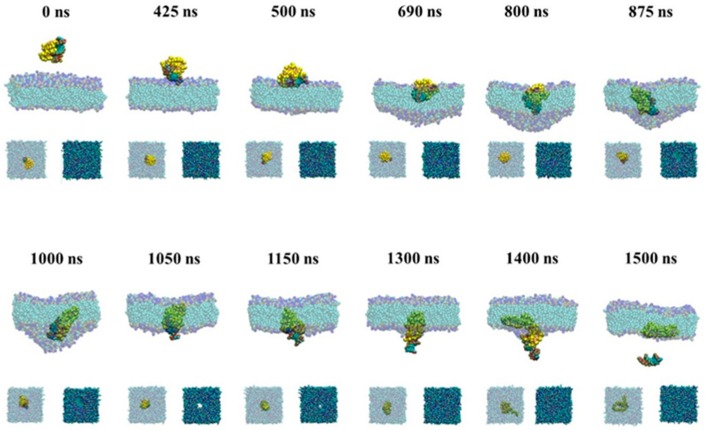
Snapshots of the active transport of siRNA assisted by PMAL. The major graphs are front view of process and the small graphs are upper view.

To give the details of the passive and active transport of the siRNA assisted by PMAL through the membrane, we give the snapshots of the passive and active transport in Figure 5 and Figure 6, respectively.

#### 2.2.1. Passive Transport of siRNA Assisted by PMAL

Figure 5 gives snapshots of the passive transport of siRNA assisted by PMAL. When siRNA-PMAL complex approaches to the surface of lipid bilayer membrane, it rotates and makes PMAL interact with membrane due to electrostatic interaction between PMAL and membrane (1150 ns). In the passive transport, PMAL has enough time to spread in the upper leaflet of membrane (1500 ns), resulting in the increase of radius of gyration of PMAL shown in Figure 4c. The extended PMAL can carrier siRNA like a boat and help siRNA enter membrane (4250 ns). Then the lipid bilayer membrane is deformed due to the insertion of siRNA-PMAL complex (7500 ns), which is consistent with results shown in Figure 4b. Due to the hydrophilicity of siRNA, PMAL entangles around siRNA in hydrophobic environment (10,000 ns), which is the nature of interior of lipid bilayer membrane. This causes the decrease of radius of gyration of PMAL as shown in Figure 4c. Finally, PMAL punches on the membrane (10,500 ns) to form a channel for siRNA delivery, as shown in supported information (Figure S4). The siRNA leaves the lipid bilayer membrane (13,250 ns), while PMAL still stays in the membrane (14,250 ns). Due to the hydrophobic interaction between PMAL and lipid tails, PMAL will stay in the lipid bilayer membrane after siRNA delivery (15,000 ns).

#### 2.2.2. Active Transport of siRNA Assisted by PMAL

Figure 6 gives snapshots of the active transport of siRNA assisted by PMAL. In the active transport, siRNA in the complex prefers to interact with the surface of membrane due to the electrostatic interaction (425 ns). Due to quick movement, the complex has no enough time to rotate and siRNA lies down and penetrates into the membrane (500 ns), which is similar with those of the naked siRNA shown in Figure 2 (500 ns). Then, the lipid bilayer membrane begins to deform at 690 ns due to the strong repulsion between siRNA and lipid tails. In this case, we believe that PMAL has little effect on assisting siRNA transport, which is consistent with results shown in Figure 4e–h. Once the siRNA-PMAL complex totally merges into the lipid bilayer membrane (800 ns), the dissociation between siRNA and PMAL starts due to stronger hydrophobic interaction between PMAL and lipid tails. When the complex moves to the lower leaflet of lipid bilayer membrane (1000 and 1050 ns), siRNA leaves the membrane first, while PMAL remains in membrane (1400 ns).

## 3. Discussion

### 3.1. Potential of Mean Force for the Naked siRNA And siRNA-PMAL Complex During Transmembrane Process

**Figure 7:**
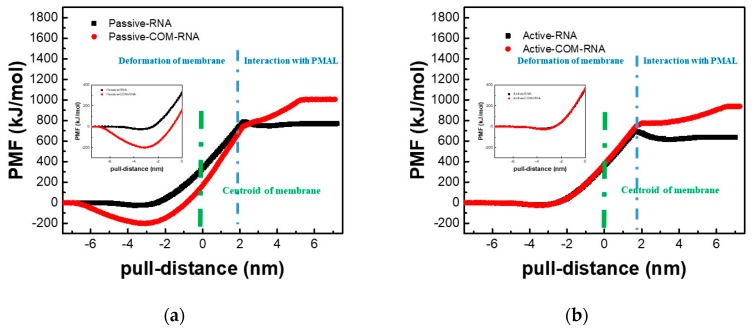
PMFs of the naked siRNA and the siRNA-PMAL complex during transmembrane. (**a**) Passive transport. (**b**) Active transport.

We further studied the transmembrane process of the naked siRNA and the siRNA-PMAL complex by means of potential of mean force (PMF), which are shown in Figure 7.

It is shown in Figure 7a,b (black lines) that for the transmembrane process of the naked siRNA, the active and passive transport display the similar behavior. When the naked siRNA gets close to the lipid bilayer membrane, the free energy slightly reduces to −21 kJ/mol and −25 kJ/mol at the position of −3.7 nm to the membrane center for the active and passive transport, respectively. Once the naked siRNA penetrates the lipid bilayer membrane, the free energy increases sharply for both the active and passive transport, indicating the difficulty of transmembrane of the naked siRNA. This is consistent with our previous simulation work [24]. It is also shown that values of the maximum force for the active and passive transport are 195 kJ·mol^−1^·nm^−1^ and 207 kJ·mol^−1^·nm^−1^, respectively. We can conclude that the transmembrane of the naked siRNA in both the active and passive mode cannot occur spontaneously. 

Figure 7 also gives PMFs of PMAL-assisted siRNA transmembrane for the active and passive transport. It is shown that there is significant difference between active and passive transmembrane of siRNA assisted by PMAL. In passive transport (Figure 7a), the PMF decreases to −200 kJ/mol at nm, indicating that the process of PMAL-assisted siRNA transmembrane in the passive transport is energy favorable and can occur spontaneously. In active transport (Figure 7b), however, the PMF curve of PMAL-assisted siRNA transmembrane is same with that of the naked siRNA. This process is not energy favorable, indicating PMAL cannot assist siRNA through the membrane in active transport mode. 

Different from the transmembrane of the naked siRNA, there is no obvious energy barrier when siRNA leaves the membrane (blue dash line in Figure 7a,b), indicating that PMAL can lower the energy barrier and help siRNA release from the membrane. However, the additional energy is necessary for the separation of siRNA and PMAL. Therefore, the properties of PMAL, including molecular weight and hydrophobic segment, should be carefully chosen for siRNA delivery, which have been extensively studied in our previous work [24,33].

### 3.2. The Possible Mechanism of siRNA Delivery Assisted by PMAL

**Figure 8:**
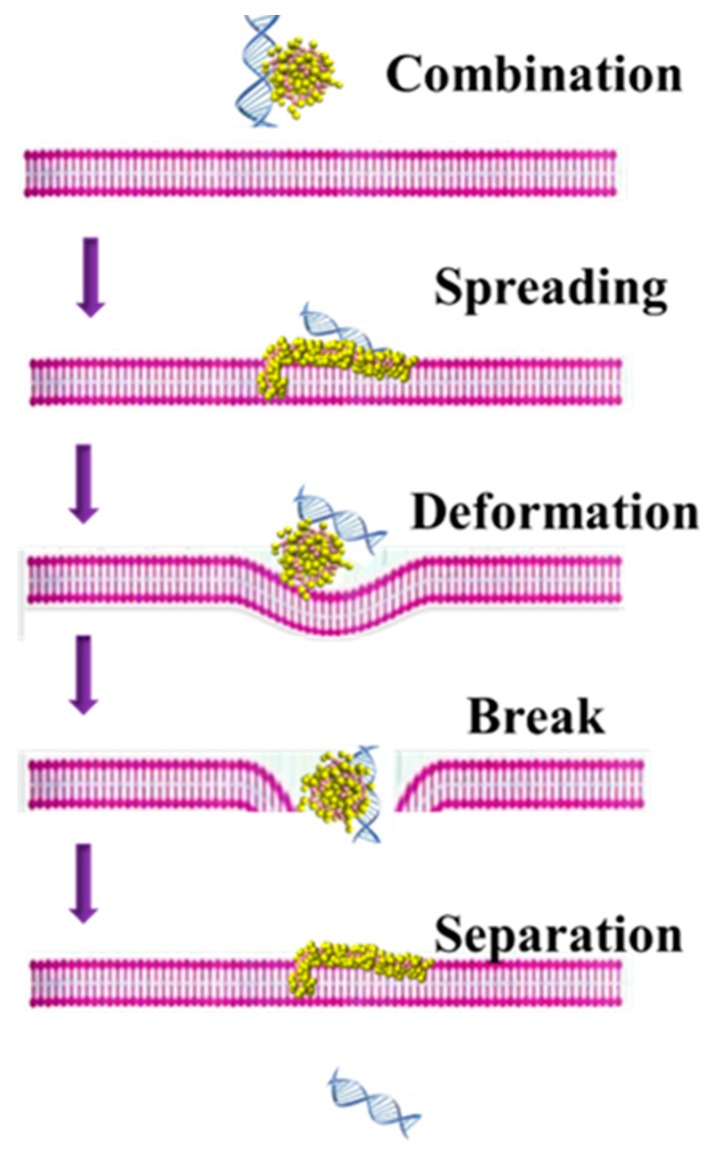
The possible mechanism of siRNA delivery assisted by PMAL.

The possible mechanism of siRNA delivery assisted by PMAL is summarized in Figure 8.

Firstly, PMAL and siRNA can form stable complex due to electrostatic interaction between the negatively charged siRNA and positively charged PMAL. When the siRNA-PMAL complex approaches the surface of lipid bilayer membrane. Then PMAL interacts with membrane in the passive transport mode and punches a hole on the membrane to help siRNA enter into the lipid bilayer. This is an energy favorable process. At the meantime, siRNA can penetrate into the membrane easily. Then the membrane deform to prevent the transport behavior and PMAL started to be squeezed. Thus PMAL entangles around siRNA and help it pass through the lipid bilayer membrane. Finally, the siRNA moves out with PMAL trapped in the membrane.

## 4. Materials and Methods

### 4.1. Models

#### 4.1.1. Coarse-Grained Model of Membrane

**Figure 9:**
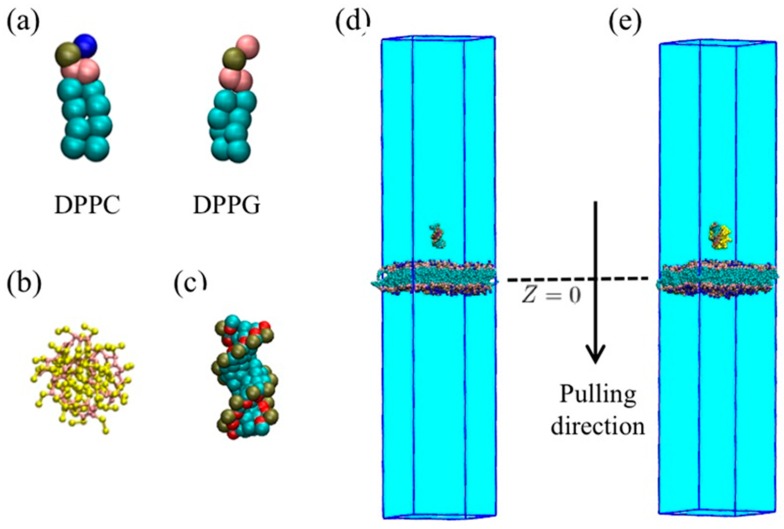
Models of lipid, siRNA, and lipid bilayer membrane. (**a**) Coarse-grained model of DPPC and DPPG, here choline group was colored in blue, phosphorus group was colored in brown, glycerol group was colored in pink and fatty acid chain was colored in cyan; (**b**) coarse-grained model of PMAL, here backbone is colored in pink, side chain is colored in yellow; (**c**) coarse-grained model of siRNA, here red beads are sugar particles, cyan beads are base particles, green beads are phosphate particles; (**d**) model of the naked siRNA transmembrane; (**e**) model of siRNA-PMAL complex transmembrane.

The general martini coarse-grained neutral DPPC (dipalmitoyl phosphatidylcholine) and negative DPPG (dipalmitoyl phosphatidylglycerol) were chosen to build the lipid bilayer membrane [34,35,36], as shown in Figure 9a. Initially, both upper and lower leaflets of the lipid bilayer membrane contained 273 DPPC and 91 DPPG molecules. The length and width of the lipid bilayer membrane were both 16 nm. The lipid bilayer membrane was located in the center of simulation box with size of 16 × 16 × 11 nm, as shown in Figure S1a. Water molecules were filled into the simulation box with the density of 1.0 g/mL. The total negative charge of the membrane is −182e, and for all the systems, counter Na^+^ cations were added to make total charge zero. Two kinds of lipids were placed randomly, and then a MD simulation with an annealing process was conducted to readjust membrane structure. The details were given in support information.

#### 4.1.2. Coarse-Grained Model of PMAL

The coarse-grained (CG) model of PMAL was built according to our previous study based on the Martini force field [24,34]. In this work, 32 units PMAL model with a total charge of +27e was chosen based our previous work [33]. Each of PMAL unit had six heavy Martini particles as shown in Figure 9b.

#### 4.1.3. Coarse-Grained Model of siRNA and Water.

The siRNA was built as our previous work [24,33]. Briefly, a sequences of 5′-CGCGAAUUCGCG -3′ was chosen to form a siRNA duplex with a total charge of −22e in a fully deprotonated state as shown in Figure 9c. Four water molecules were treated as one coarse-grained bead with a mass of 72 amu.

#### 4.1.4. Complex of PMAL and siRNA

PMAL and siRNA were centered into simulation box of 12 × 12 × 12 nm in size as shown in Figure S2a. Then a MD simulation with annealing process was conducted to generate siRNA-PMAL complexes with total a charge of +5e as shown in support information. We chose complexes of equilibrium from the independent conformations as initial states for further steered molecular dynamics. For all the systems, counter cations were added to make total charge zero.

#### 4.1.5. Transmembrane Model

The naked siRNA and PMAL-siRNA complex were discussed separately. The lipid bilayer membrane was placed at the center of simulation box with a size of 16 × 16 × 80 nm. The naked siRNA or PMAL-siRNA complex were set above the surface of membrane with distance of 2 nm, as shown in Figure 9d,e. Counter NA cations were added to neutralize the charge of the system. Then a 400 ns short simulation was conducted to equilibrate the simulation system.

### 4.2. Simulation Details

All simulations were conducted with MD engine GROMACS (Version 4.5.4) (Herman Berendsen’s group, Department of Biophysical Chemistry of Groningen University, Groningen, Holland) [37] and VMD software (NIH Biomedical Research Center for Macromolecular Modeling and Bioinformatics, Urbana, IL, USA) [38] was used to visualize snapshots.

#### 4.2.1. Transmembrane Simulation

The initial structure was built as mentioned above, then a 400 ns equilibrium simulation was conducted using NPT ensemble. The temperature and the pressure were set as 310 K and 1 bar using the v-rescale and Parrinello–Rahman method, respectively [39,40]. In all simulations, the pressure was controlled in semi-isotropic mode with the compressibility of 3 × 10^–4^. Lenard–Jones potential was shifted smoothly from 0.9 nm to 1.2 nm. The electrostatic interaction was processed with a cut-off of 1.2 nm, the potential was shifted to 0 from 0.0 nm to cut-off distance. All the simulations were performed at a time-step of 20 fs. The position of siRNA was restricted in the equilibrium process. After equilibrium, a steered MD simulation was used to reveal the transmembrane process. The constant pulling force (2000 kJ·mol^−1^·nm^−2^) was applied to perform the steered MD simulation with constant velocity along Z direction. For both the naked siRNA and the siRNA-PMAL complex, the pulling forces were applied to the mass center of siRNA. Two pulling rates were chosen, namely the slow rate of 10^−6^ nm·ps^−1^ and the quick rate of 10^−5^ nm·ps^−1^, representing the passive transport and the active transport, respectively. The simulation time for the passive transport and the active transport of the naked siRNA or siRNA-PMAL complex were 15 μs and 1.5 μs, respectively.

#### 4.2.2. PMF Calculation

The potential of mean force (PMF) was obtained using umbrella sampling and the WHAM method [41,42]. Briefly, a series of conformations with a width of 0.1 nm that obtained from the steered MD simulation along reaction coordinate was chosen for 200 ns additional constraint equilibrium simulation with a time-step of 20 fs. Other simulation parameters were same with those in the steered MD simulation and the histograms of configuration within the umbrella sampling windows are shown in support information (Figure S5).

### 4.3. Analytical Methods

#### 4.3.1. Radius of Gyration (*R*_g_)

The radius of gyration (*R*_g_) was calculated by means of the g_gyrate command in GROMACS program. The equation is

(1) $$R_{g}={\left(\frac{\sum_{i}{\left\|r\right\|}_{i}{}^{2}m_{i}}{\sum_{i}m_{i}}\right)}^{\frac{1}{2}}$$

where *m_i_* is the mass of coarse-grained bead *i*, and *r_i_* is the position of bead *i* respect to the mass center of PMAL molecule.

#### 4.3.2. Contact Area (*C*_A_)

To get the contact area (*C*_A_) between two molecules, the solvent accessible surface areas (*SAS*) of two molecules and their corresponding complex were calculated by means of the g_sas command in GROMACS program via solvent probe with the radius of 0.14 nm. Then *C*_A_ was calculated as

(2) $$C_{A}=\frac{SAS_{1}+SAS_{2}-SAS_{C}}{2}$$

where *SAS*_1_, *SAS*_2_, and *SAS*_C_ are the solvent accessible surface areas of molecule 1, molecule 2, and their corresponding complex, respectively.

#### 4.3.3. Order Parameter of Lipid (*P*_2_)

To describe the flatness of lipids in the membrane, the order parameter (*P*_2_) of lipid molecule is defined as

(3) $$P_{2}=\langle \frac{3\cos^{2}\theta -1}{2}\rangle$$

where *θ* is the angle between the bond of tail particle of lipid molecule and the normal of bilayer lipid membrane. *P*_2_ is the bond order parameter of lipid molecule, which is the average of all bond order parameters of lipid molecules in bilayer membrane. *P*_2_ = 1 means all lipid molecules are well aligned with the normal direction of bilayer lipid membranes; *P*_2_ = −0.5 is anti-aligned; and *P*_2_ = 0 means random orientation of lipid molecules in membrane.

## 5. Conclusions

In this study, the active and passive transport of siRNA were discussed using steered molecular dynamics simulations based on the martini coarse-grained model. The siRNA transmembrane mechanism assisted by PMAL has been further elucidated. For the transmembrane of the naked siRNA, there is no significant difference between the active and passive transport. The naked siRNA transmembrane processes in both the active and passive transport are free energy unfavorable, indicating these processes cannot occur spontaneously. With the help of PMAL, there is significant difference between the passive and active transport. It is demonstrated that the passive transport is the main way for PMAL-assisted siRNA transmembrane via PMF, structure, and energy analysis. In the active transport, PMAL has no effect on siRNA transmembrane compared with transmembrane of the naked siRNA. The structure of membrane is destroy by PMAL. First, the membrane deformation caused by PMAL-siRNA complex during transmembrane process due to the interaction between PMAL and membrane. Secondly, after siRNA is delivered through the membrane, PMAL is still in the membrane. As discussed above, PMAL shows the potential in application of siRNA delivery. The underlying mechanism is elucidated for PMAL-assisted siRNA transmembrane. The delivery of siRNA with PMAL should be a passive transport. PMAL can help siRNA insert into the membrane and hold a channel for transport. In the future, we can further design new PMAL molecules to help siRNA delivery by carefully tuning their interactions with lipid bilayer membrane and the target siRNA.

## Supplementary Materials

The following are available online at http://www.mdpi.com/1420-3049/23/7/1586/s1, Figure S1: The lipid bilayer membrane before and after an annealing MD simulation [43,44]. Figure S2: The formation of stable siRNA-PMAL complex using an anneal MD simulation. Figure S3: The changes of lipid bilayer membrane during the passive and active transport of siRNA assisted by PMAL. Figure S4: The channel formation of the siRNA delivery assisted by PMAL. Figure S5: The histogram of configurations within the umbrella sampling windows. Figure S6. Flatness of membrane for naked siRNA.
