# Supplementary material for: The Mechanism for siRNA Transmembrane Assisted by PMAL
Source: Molecules. 2018 Jun 29;23(7):1586. doi: 10.3390/molecules23071586 (PMC6099945; doi:10.3390/molecules23071586)
Supplement: Supplementary file 1 [file molecules-23-01586-s001.pdf]

molecules

MDPI

Article

The Mechanism for siRNA Transmembrane Assisted by PMAL

Yanfei Lu [1], Jipeng Li [2], Nan Su [2] and Diannan Lu [2,*]

Department of Chemical Engineering, Tsinghua University, Beijing, 100084, China; lu511966207@163.com (Y.L.); lijipeng0927@163.com (J.L.); sunan12345@126.com (N.S.)

* Correspondence: ludiannan@tsinghua.edu.cn; Tel.: +86-010-62783153

Received: 16 May 2018; Accepted: 19 June 2018; Published: date

Supporting information

1. Models and simulation methods

1.1. Annealing simulation

The simulation system is shown in Figure S1(a). The annealing process was performed in NPT ensemble. Pressure was set as 1 bar using Parrinello-Rahman method [1] in isotropic mode (semi-isotropic for membrane system) and the value of compressibility was set as 3×10^{-4}. The v-rescale method [2] was used to control the temperature as following protocol in each cycle. Firstly, temperature increased from 300 K to 360 K during first 50 ns and maintained at 360 K for 50 ns, then it decreased to 300 K in next 80ns and maintained for 20 ns. The total annealing process contained 10 circles and the total simulation time was 2 μs. After annealing simulation, additional traditional MD simulation was conducted for 1μs at 310 K. In annealing and traditional MD simulation, the time-step was set as 20 fs. Lenard-Jones potential was shifted smoothly from 0.9 nm to 1.2 nm. The electrostatic interaction was processed with a cut-off of 1.2 nm, the potential was shifted to 0 from 0.0 nm to cutoff distance.

1.2. Stabilization of membrane

As mentioned above, the temperature is shown in Figure S1(b). The area of lipid bilayer changes during annealing process and shows positive correlation with temperature (the inner graph in Figure S1(c)). There is no difference in the density of lipids before and after annealing process, as shown in Fig S1(d). This indicates that lipids hardly moved along Z direction. Moreover, the lateral diffusion constant (D) was calculated by mean square displacement (MSD) as shown in Figure S1(e). The value of DPPC is 6.32×10^{-7} cm^2/s which is only slightly smaller than DPPG, 6.40×10^{-7} cm^2/s, which is comparative to the experimental result [3,4]. The favorable linearity of MSD and stable area in the last 1μs equilibrium simulation suggest the steadiness of the final membrane structure after annealing.

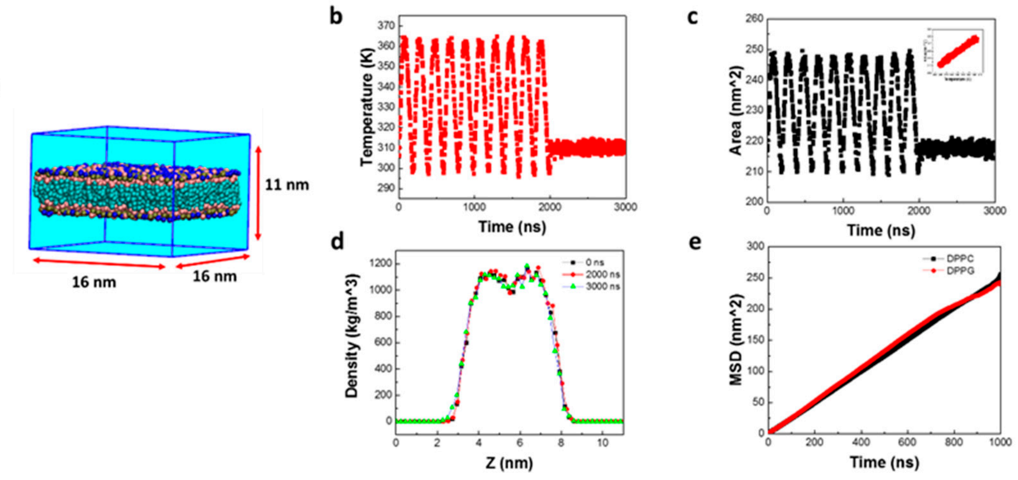

Figure S1. The lipid bilayer membrane before and after an annealing MD simulation. [(a) The model of bilayer membrane; (b) Temperature of the annealing process; (c) The area of membrane; (d) The density of membrane along Z direction; e) MSD of lipids.

1.3. The complexation of PMAL and siRNA

As mentioned above, annealing MD simulation process was used to find stable structure of the siRNA-PMAL complex as shown in Figure S2. The change of temperature was shown in the inner graph of Figure S2(b). The total interaction energy between PMAL and siRNA decreased form –765±129 kJ/mol to –926±71 kJ/mol, indicating the formation of the stable siRNA-PMAL complex. The contact area between siRNA and PMAL increases from 6.31±1.05 nm^2 to 7.45±0.67 nm^2 as shown in Figure S2(c). Therefore, a collection of optimized stable siRNA-PMAL complexes was obtained after the annealing MD simulation.

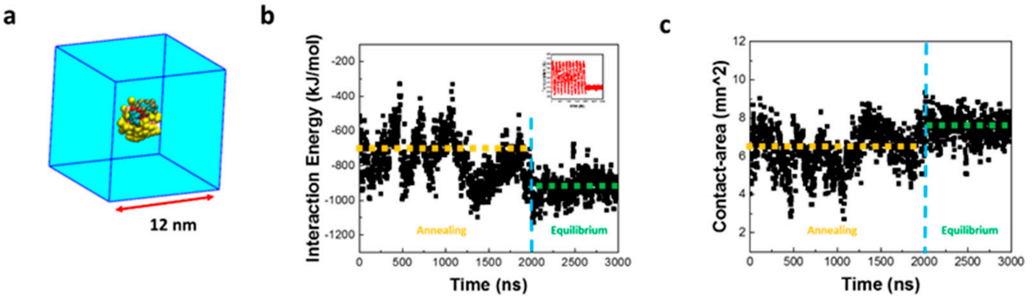

Figure S2. The formation of stable siRNA-PMAL complex using an anneal MD simulation. (a) The smodel of complex; (b) The interaction between PMAL and siRNA; (c) The contact area of PMAL and siRNA.

2. Transmembrane process of siRNA assisted by PMAL

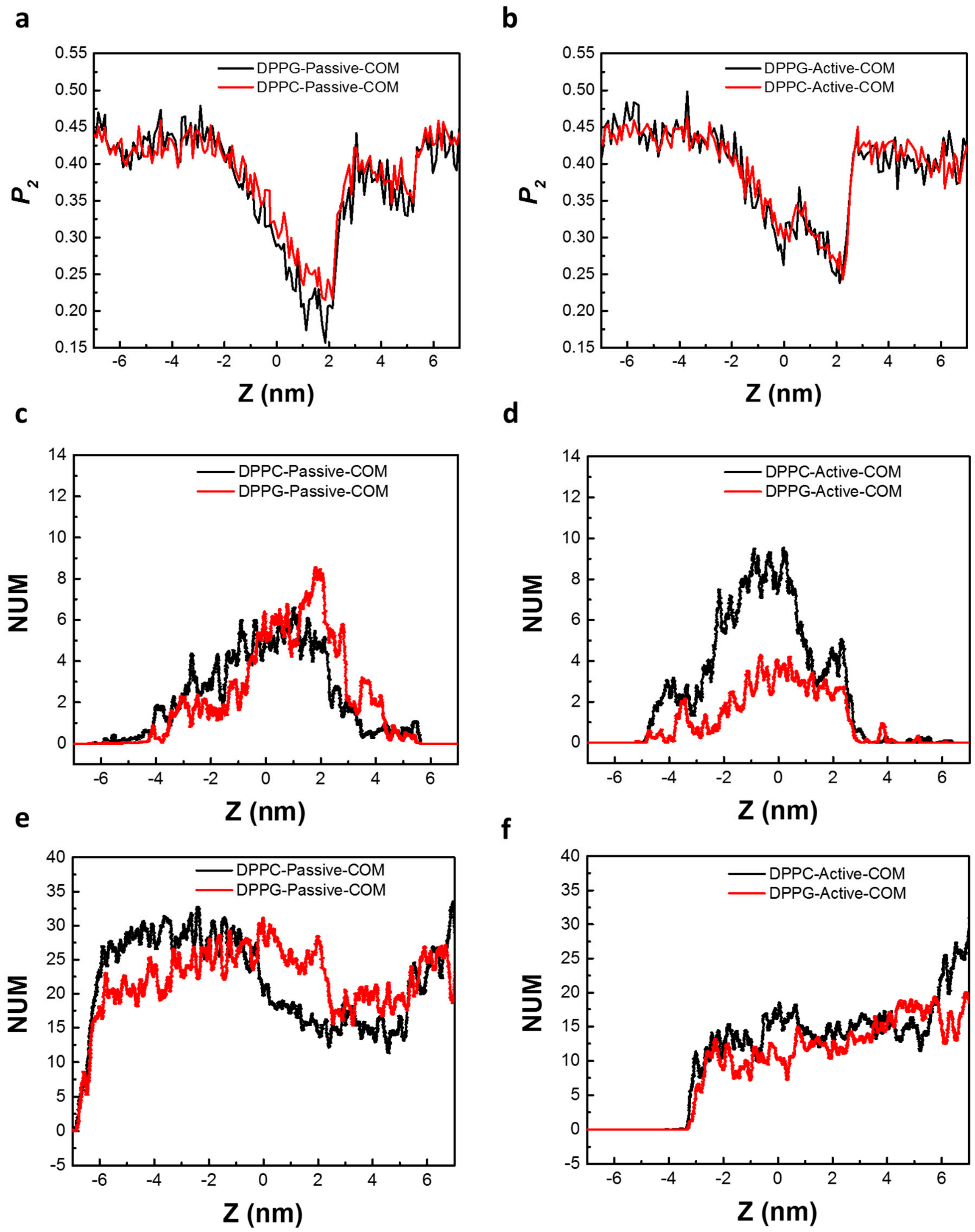

Figure S3. The changes of lipid bilayer membrane during the passive and active transport of siRNA assisted by PMAL. (a) The changes of the order parameter, P2, in passive transport; (b) The changes of the order parameter, P2, in active transport; (c) The lipid number around siRNA in passive transport; (d) The lipid number around siRNA in active transport; (e) The lipid number around PMAL in passive transport; f) The lipids number around PMAL in active transport.

3. Transmembrane process of siRNA assisted by PMAL

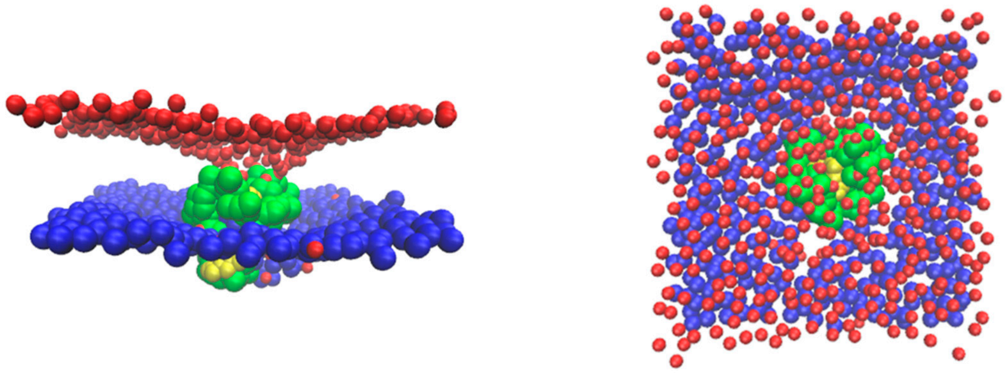

Figure S4. The channel formation of the siRNA delivery assisted by PMAL. The red particles are PO4 groups of upper layer, the blue particles are PO4 groups of lower layer, the green particles are PMAL and the yellow particles are siRNA.

4. Potential of mean force for the naked siRNA and siRNA-PMAL complex during transmembrane process

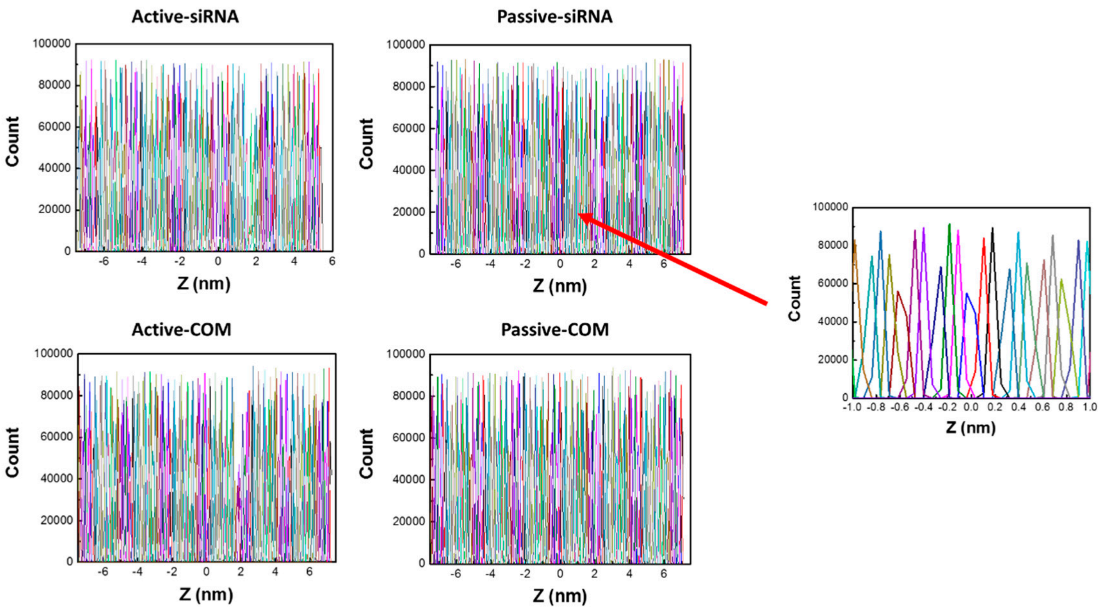

Figure S5. The histogram of configurations within the umbrella sampling windows.

As shown in Figure S5, the width of window in umbrella sampling is 0.1 nm along the reaction coordinate and the histograms suggest that there is sufficient overlap between adjacent windows.

5. Flatness of membrane for naked siRNA

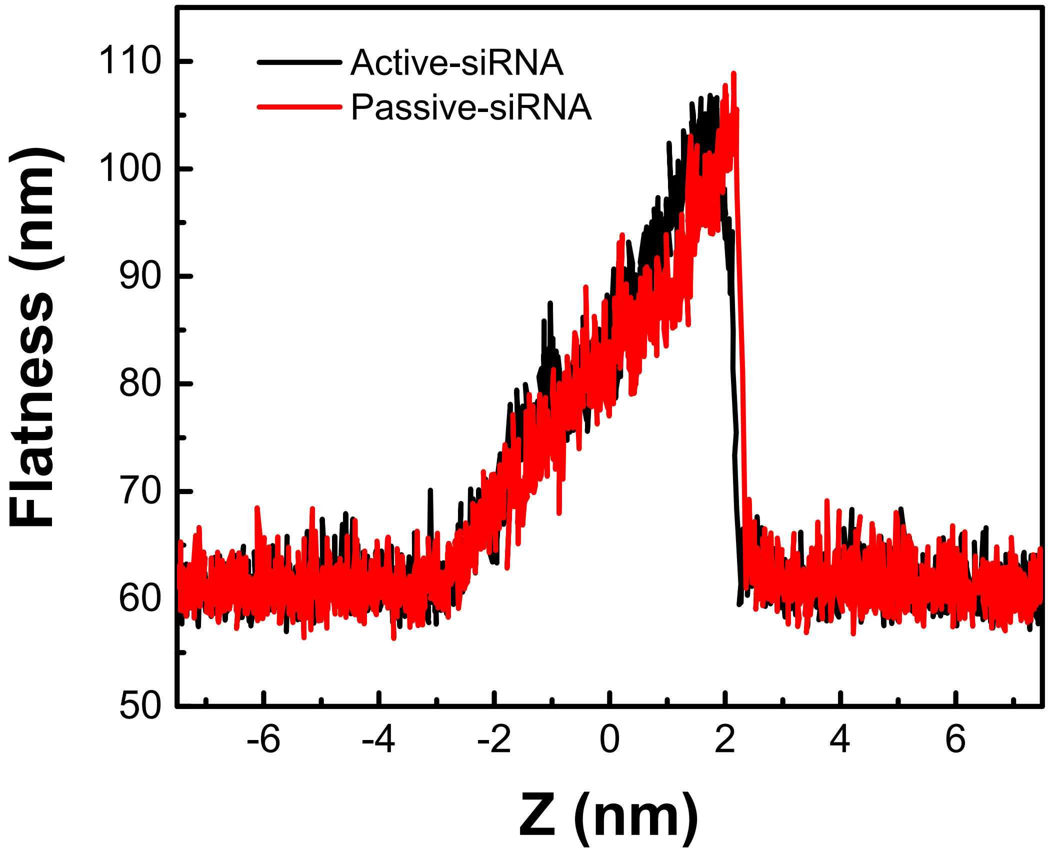

Figure S6. Flatness of membrane for naked siRNA. The flatness of membrane was calculated by the max Z value minus the min Z value of membrane.

References

1. Parrinello, M.; Rahman, A.; Polymorphic transitions in single-crystals - a new molecular-dynamics method. J. Appl. Phys. **1981**, *52*, 7182–7190.
2. Bussi, G.; Donadio, D.; Parrinello, M. Canonical sampling through velocity rescaling. J. Chem. Phys. 2007, 126.
3. Gabriel, B.; Teissie, J. Proton lateral conduction along lipid monolayers is present only in the liquid-expanded state. J. Am. Chem. Soc. **1991**, *113*, 8818–8821.
4. Tamm, L.K.; Mcconnell, H.M. Supported phospholipid bilayers. Biophys. J. **1985**, *47*,105.
